# Visual acuities as measured with an automatic intelligent visual acuity chart projector and standard logarithmic visual acuity chart: a prospective comparative study

**DOI:** 10.3389/fmed.2025.1447679

**Published:** 2025-02-24

**Authors:** Mingyue Luo, Xinyu Liu, Dingding Zhang, Youxin Chen, Weijuan Kang

**Affiliations:** ^1^Department of Ophthalmology, Peking Union Medical College Hospital, Chinese Academy of Medical Sciences and Peking Union Medical College, Beijing, China; ^2^Beijing Key Laboratory of Fundus Diseases Intelligent Diagnosis & Drug/Device Development and Translation, Beijing, China; ^3^Key Laboratory of Ocular Fundus Diseases, Chinese Academy of Medical Sciences, Beijing, China; ^4^Center for Prevention and Early Intervention, National Infrastructures for Translational Medicine, Institute of Clinical Medicine, Peking Union Medical College Hospital, Chinese Academy of Medical Science and Peking Union Medical College, Beijing, China

**Keywords:** standard logarithmic visual acuity chart, intelligent projector visual acuity chart, visual acuity, repeatability analysis, consistency analysis

## Abstract

**Purpose:**

This study aimed to test the repeatability of visual acuity (VA) measurement using an novel intelligent projector visual acuity (IP) chart (LSJ-IVAC-6000A, Hunan Liangshi Jia Biotechnology Co., Ltd.) and the consistency of VA measurement between the IP chart and the 5 m standard logarithmic visual acuity (SL) chart (GB11533-2011).

**Methods:**

In this prospective comparative study, 53 subjects were included to test the internal repeatability of the IP chart. Both eyes of the subjects were tested three times, with a minimum interval of 10 min. The intraclass correlation coefficient (ICC) was used to evaluate the repeatability. One hundred subjects were included to test the consistency between two charts. Both eyes of the subjects were tested with two charts in random order with an interval of 10 min for each test. ICC and the Bland–Altman statistical analyses were used to analyze the consistency of the two charts. VA values were expressed by the logMAR recording method. The time consumption for each test was recorded in seconds.

**Results:**

In the repeatability analysis, the ICC values of the right and left eye were 0.968 (95% CI, 0.950–0.981, *p* < 0.001) and 0.964 (95% CI, 0.944–0.978, *p* < 0.001), respectively. In the consistency analysis, the ICC values of the right and left eye were 0.946 (95% CI, 0.919–0.963, *p* < 0.001) and 0.817 (95% CI, 0.727–0.877, *p* < 0.001), respectively. However, the 95% limits of agreement (LoA) of the differences in the VA measurements between the IP and SL chart for the right and left eye were −0.26 to 0.31 and −0.34 to 0.39, respectively, suggesting a certain degree of instability in the measurements of the SL chart. There was no significant difference in the time consumption of VA measurements between the two charts (*p* = 0.668). In the consistency analysis of subgroups by age, the ICC values were >0.8 (*p* < 0.001) in the most groups except for the left eyes of 51–70 years old subgroup which had an ICC value of 0.448 (95% CI, −0.919 to 0.841, *p* = 0.17), with 95% LoA of −1.02 to 0.77.

**Conclusion:**

The IP chart demonstrated good repeatability and overall consistency with the SL chart.

## Introduction

Visual acuity (VA) measurement is at the core of clinical practice and research in ophthalmology. The Early Treatment Diabetic Retinopathy Study (ETDRS) chart, which provides accurate and repeatable measurements of VA, is considered the gold standard in international clinical research (1). However, the time-consuming procedures and inability of some examinees to read “SLOAN” letters limit the use of ETDRS charts for VA measurement in China (2). Instead, the 5 m standard logarithmic visual acuity (SL) chart (GB11533-2011) (3–5) is widely adopted in China in public screening and clinical practice. It was designed in 1959 and revised in 2011. The optotype E is used with an increment rate of ^10^√10 by line with 24 mm line spacing. Each optotype is a squared “E” with all paths equal in width and length, rotated 90 degrees in horizontal and vertical orientations (5). The SL charts are usually set at a 5 m distance with standardized illumination (2, 4), as compared to a 6 m distance most recognized internationally (6).

Over the years, the accuracy and reliability of SL chart has been widely proven, although there are some limitations (2). First, the optotypes on the chart are fixed, which are easy to memorize. Second, the degradation of optotypes and decreased light intensity after long-term uses of the light box, affect measurement results. Third, the 5 m SL chart occupies a large space, bringing out derivatives as SL charts set at 2.5 m, compromising the accuracy of VA measurement for those with reserved accommodation (7). In addition, there must also be a committed staff to conduct VA measurements, thus increasing manpower costs.

In recent years, automated and self-administered visual acuity (VA) tests have garnered increasing attention due to their potential to facilitate vision assessment without the involvement of eye health professionals. These methods leverage digital platforms, enabling patients or non-specialists to monitor visual function remotely, thereby augmenting teleophthalmology and other remote healthcare services. Such innovations address key challenges in traditional VA testing, including the need for in-person clinical administration and the strain on limited ophthalmology resources. Furthermore, these technologies empower patients with chronic eye conditions to conduct autonomous, frequent monitoring, reducing the frequency of clinic visits. Pragmatic trials have demonstrated that certain self-administered VA tests can achieve accuracy comparable to clinical assessments in controlled setting (8).

**Figure 1:**
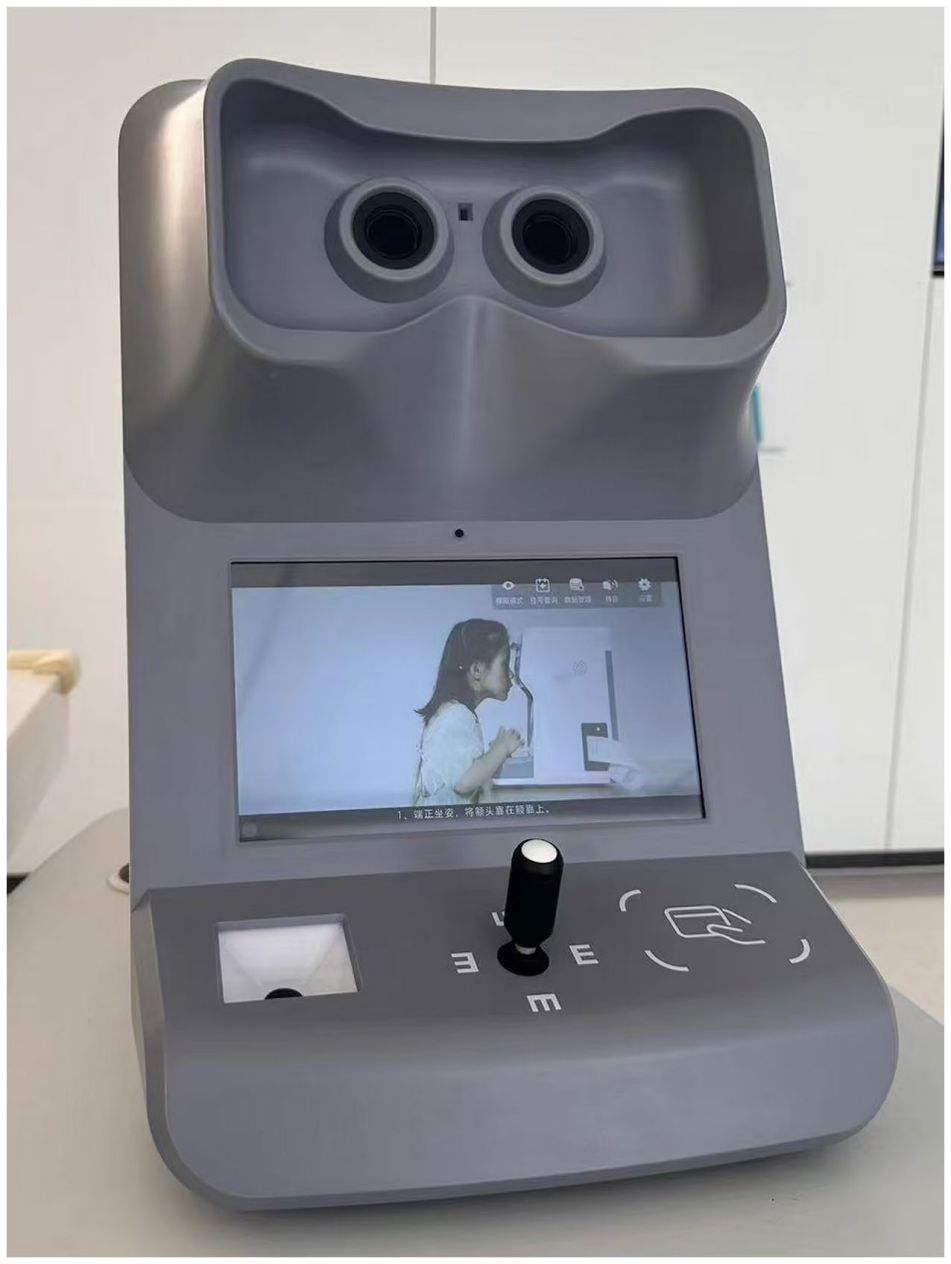
Photograph of the intelligent projector visual acuity (IP) chart (LSJ-IVAC-6000A, Hunan Liangshi Jia Biotechnology Co., Ltd.).

Building on these advancements, an intelligent projector visual acuity (IP) chart (LSJ-IVAC-6000A, Hunan Liangshi Jia Biotechnology Co., Ltd.) was designed to overcome the drawbacks of SL VA measured by a traditional light box chart. It is a fully automatic self-administered vision testing device (Figure 1), designed in accordance with the national standard GB11533-2011 (4). The purpose of this study is to evaluate the repeatability of visual acuity assessment using the IP chart, as well as the consistency of visual acuity measurement between the SL chart and the IP chart.

## Methods

This was a prospective, comparative study. The study was approved by the Institutional Review Board (K-4143) of Peking Union Medical College Hospital and was conducted in accordance with the tenets of the Declaration of Helsinki. Written informed consent was obtained from each subject or guardian if the subject was under age 18.

### Subjects

Subjects were recruited from patients and their accompanying persons in routine ophthalmology clinics, as well as healthy volunteers from nearby communities. Inclusion criteria include: (1) age between 6–70 years; (2) capable of cooperating with visual acuity examinations. Exclusion criteria include: (1) VA worse than 0.05 decimal VA measured by SL chart (which is equivalent to 1.3 logMAR VA), representing the lowest VA measurable by the intelligent visual acuity chart projector; (2) inability to operate the projector.

The sample size calculation was conducted using PASS 2021, v21.0.3. For the repeatability analysis of the IP chart, based on a preliminary trial with 29 participants, we estimated an intraclass correlation (ICC) of 0.83 and determined that a two-sided 95% confidence interval would have a width of 0.1427. Consequently, when each subject was measured three times, a random sample of 53 subjects was found to be necessary to achieve the stated confidence interval. For consistency analysis, from a pre-experiment involving 29 participants, we obtained the mean and standard deviation of the differences between the IP chart and the SL chart as −0.0013 logMAR and 0.085 logMAR, respectively. With a type I error (*α*) set at 0.05 and a type II error (*β*) at 0.10, and defining the pre-defined clinical agreement limits (*δ*) as (−0.22, 0.22) logMAR, we determined that a total sample size of 96 participants is necessary to achieve 90% power for assessing agreement between the two measurement methods.

### Visual acuity chart and measurement

Subjects were tested with spectacles if they were routinely worn.

When tested with the IP chart, the examinee rests their forehead against the device and look forward. The device projects optotypes based on optical imaging design of GB11533-2011 (4) with a background brightness of 200 cd/m^2^, ensuring consistency with traditional vision charts. Briefly, the build-in liquid crystal display (LCD) projects the optotype as a virtual image at 5 m distance through converging lenses. The LCD screen works separately when testing different eyes. The optotype is only projected to the tested eye, while a blank screen is shown to the non-tested eye. In this way, the eyes can be tested separately. The examinee uses a joystick to select the direction of the optotype, and the built-in algorithm simulates the judgment of clinical optometrists according to the GB11533-2011 (4), evaluates each response of the examinee, adjusts optotypes accordingly, the visual acuity measured by the new IP chart was scored based on the number of smallest optotypes correctly recognized, with the number exceeding half of the total optotypes in the corresponding row of the SL chart at the same visual acuity level. SL chart was used to acquire VA in compliance with the requirements defined in GB11533-2011 (4). A single experienced optometrist made the measurements. The examinee sits at a distance of 5 m from the acuity chart and looks straight, and each eye is tested sequentially with the other eye covered by an adhesive patch. The right eye was first examined followed by the left eye. The examiner designates an optotype from the first row, and the examinee indicates the direction. If none could be read, the viewing distance was decreased to 4, 3, and 2.5 m in sequence, and the subject was again asked to identify the optotypes on the top line of the chart until all optotypes on the top line of the chart were identified correctly. The visual acuity was recorded as 0.08, 0.06, and 0.05 accordingly. If all the directions of optotypes from the first row are correct at 5 m, the examiner moves to the next row and each subsequent row. VA was scored as the smallest row where the number of optotypes correctly recognized was more than half of the total number of optotypes on the line.

### Procedure

To test the internal repeatability of the IP chart, the subjects were tested three times, with a minimal interval of 10 min. The beginning and ending time points for each test were recorded in seconds to ensure enough resting time.

To examine the consistency of the two charts, subjects were tested with the SL and IP charts. The order of the tests was determined by random number sequence, with an interval of 10 min for each test. The examiners using the SL chart were masked to the results of the IP chart if the subject was examined with the IP chart first. The start and end times of each examination were recorded in seconds.

### Statistical analysis

Data analysis was performed using visual acuities determined with the automatic visual acuity chart projector and the logarithmic visual acuity chart was converted to a logarithm of the minimal angle of resolution (logMAR). The repeatability of the IP chart was evaluated by ICC analyses. The consistency between the IP chart and SL chart was evaluated by ICC and Bland–Altman statistical analyses. The ICC was calculated using IBM SPSS Statistics V.26.0 (IBM). The Bland–Altman statistical analyses were performed using SAS JMP V.15.1 (SAS Institute).

## Results

In total, 53 subjects were included to test the repeatability of the IP chart and 100 subjects were included to test the consistency of the two charts.

In the repeatability analysis, both eyes of each subject underwent three times VA measurements using the IP chart. The ICC values of the right and left eye were 0.968 (95% CI, 0.950–0.981, *p* < 0.001) and 0.964 (95% CI, 0.944–0.978, *p* < 0.001), respectively. The ICC values for both eyes were >0.8, indicating good repeatability of the IP chart.

To further evaluate the repeatability of the measurements, Bland–Altman analyses were conducted for the first test versus the second test and the first test versus the third test. For the right eye, the mean differences were 0.03 ± 0.11 [95% limits of agreement (LoA): −0.18 to 0.24] for the first versus second test, and 0.03 ± 0.10 (95% LoA: −0.17 to 0.23) for the first versus third test. For the left eye, the mean differences were 0.03 ± 0.10 (95% LoA: −0.16 to 0.24) for the first versus second test, and 0.05 ± 0.09 (95% LoA: −0.14 to 0.23) for the first versus third test. The results showed minimal differences between the tests and narrow LoA, confirming the high stability and repeatability of the measurements.

As for time consumption, subjects used an average of 52.9 ± 15.2 s, 42.1 ± 14.3 s, and 40.6 ± 13.6 s to complete the first, second, and third test. Paired *t*-test revealed a 10.7 s and 12.3 s (both *p* < 0.05) reduction in the second and third time compared to the first time, while despite an average reduction of 1.5 s in the third time compared to the second time, the difference was not statistically significant (*p* = 0.287).

**Figure 2:**
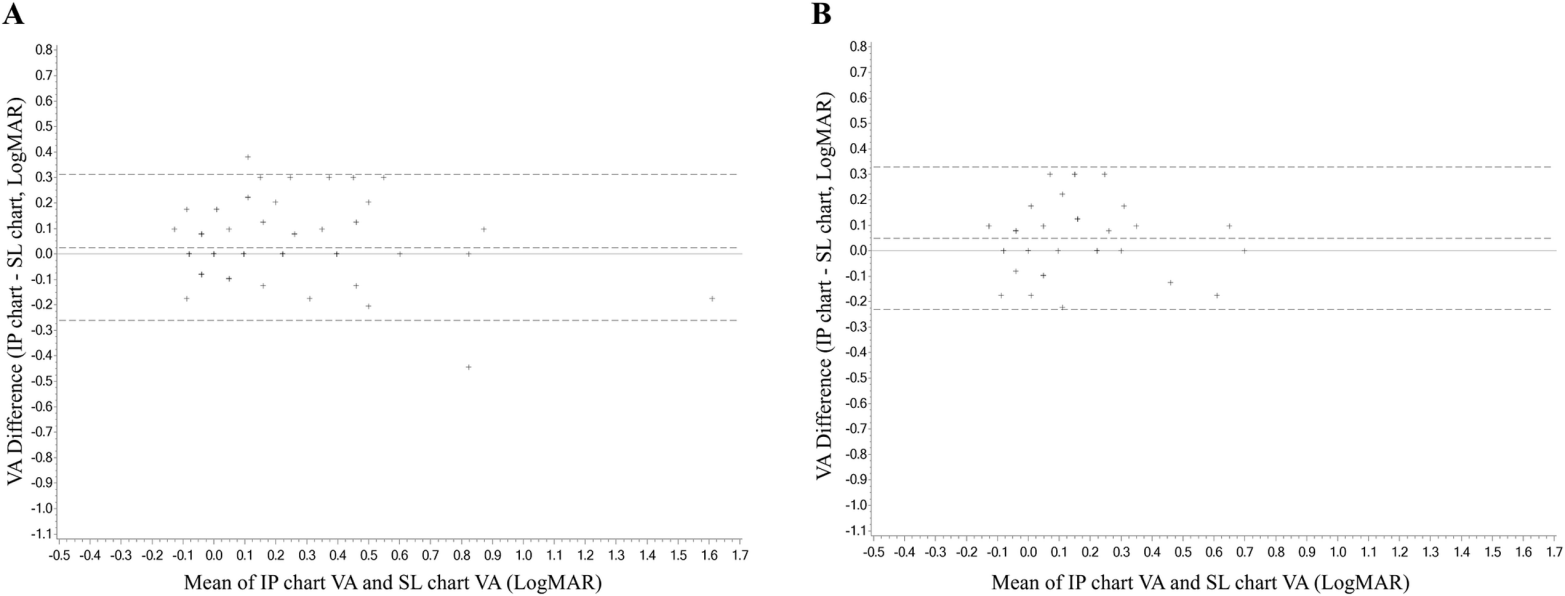
The Bland–Altman analyses diagram of the consistency between the VA measurement of the IP chart and SL chart. **(A)** Right eye. **(B)** Left eye. VA, visual acuity; logMAR, logarithm of the minimum angle of resolution; IP chart, intelligent projector visual acuity chart; SL chart, 5 m standard logarithmic visual acuity chart.

In the consistency analysis, both eyes of each subject underwent VA measurements using the IP chart and SL chart. The ICC values of the right and left eye were 0.946 logMAR (95% CI, 0.919–0.963, *p* < 0.001) and 0.817 (95% CI, 0.727–0.877, *p* < 0.001), respectively. The ICC values for both eyes were >0.8, indicating good consistency between the IP chart and the SL chart. The mean differences in VA measurements between the IP and SL chart for the right and left eye were 0.03 ± 0.12 and 0.03 ± 0.16 logMAR, respectively, with 95% LoA of −0.26 to 0.31 and −0.34 to 0.39, respectively (Figure 2). The proportions within the 95% LoA were 98.00 and 98.00%, respectively. As for time consumption, subjects used an average of 47.9 ± 14.5 and 48.6 ± 16.6 s to complete the VA measurements of the IP chart and SL chart, respectively and the paired *t*-test failed to reveal a significant difference between the two charts (*p* = 0.668).

**Figure 3:**
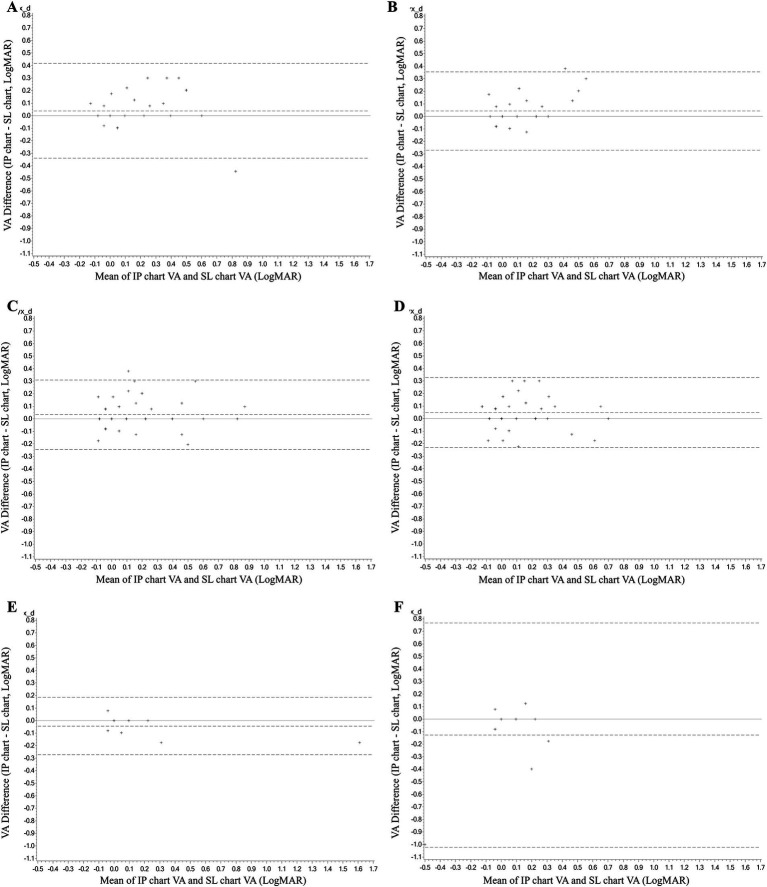
The Bland–Altman analyses diagram of the consistency between the VA measurement of the IP chart and SL chart in subgroups by age. **(A)** The right eye of the 6–18 years old. **(B)** The left eye of the 6–18 years old. **(C)** The right eye of 19–50 years old. **(D)** The left eye of 19–50 years old. **(E)** The right eye of 51–70 years old. **(F)** The left eye of 51–70 years old. VA, visual acuity; logMAR, logarithm of the minimum angle of resolution; IP chart, intelligent projector visual acuity chart; SL chart, 5 m standard logarithmic visual acuity chart.

The consistency analysis was conducted in subgroups by age (see Table 1). Six subgroups were set, containing the right and left eyes of the 6–18 years old, 19–50 years old, and 51–70 years old, respectively. The ICC values in most subgroups were >0.8 (*p* < 0.001), except for the left eyes of the 51–70 years old subgroup which was 0.448 (95% CI, −0.919 to 0.841, *p* = 0.17). It also had the largest mean difference in VA measurements between the IP and SL chart which was −0.1 ± 0.3 logMAR, with 95% LoA of −1.02 to 0.77. The mean differences in other subgroups were quite small (Figure 3).

**Table 1:** The consistency analysis of the IP chart and the SL chart in subgroups by age.

Age (years)	Side	ICC analysis			Bland–Altman statistical analyses		
		ICC value	95% CI	*p*-value	Differences between the IP and SL chart	95% LoA	Proportions within the 95% LoA
6–18 (*n* = 32)	OD	0.904	[0.804, 0.953]	<0.001	0.04 ± 0.15	[−0.34, 0.42]	96.88%
	OS	0.877	[0.748, 0.940]	<0.001	0.04 ± 0.12	[−0.27, 0.35]	96.88%
19–50 (*n* = 56)	OD	0.937	[0.893, 0.963]	<0.001	0.03 ± 0.12	[−0.24, 0.31]	98.21%
	OS	0.908	[0.843, 0.946]	<0.001	0.05 ± 0.12	[−0.23, 0.33]	100.00%
51–70 (*n* = 12)	OD	0.993	[0.975, 0.998]	<0.001	−0.04 ± 0.08	[−0.27, 0.18]	100.00%
	OS	0.448	[−0.919, 0.841]	0.17	−0.1 ± 0.31	[−1.02, 0.77]	100.00%

ICC, intraclass correlation coefficient; LoA, limits of agreement; CI, confidence interval; IP chart, intelligent projector visual acuity chart; SL chart, 5 m standard logarithmic visual acuity chart.

## Discussion

The VA measurement with a traditional light box can be influenced by various factors, including light intensity, contrast, and the degradation of optotypes (9). The design of visual acuity charts and the exam protocol also affect the results (10–13). To overcome these drawbacks and to save human and material resources, a novel intelligent projector chart was designed. In this study, we evaluated the repeatability of the IP chart and its consistency with the SL chart. The repeatability analysis yielded ICC values of 0.968 (95% CI, 0.950–0.981, *p* < 0.001) for the right eye and 0.964 (95% CI, 0.944–0.978, *p* < 0.001) for the left eye. The consistency analysis showed ICC values of 0.946 (95% CI, 0.919–0.963, *p* < 0.001) for the right eye and 0.817 (95% CI, 0.727–0.877, *p* < 0.001) for the left eye. The mean differences in VA measurements between the IP and SL charts were 0.03 ± 0.12 logMAR for the right eye and 0.03 ± 0.16 logMAR for the left eye. These results demonstrate excellent internal repeatability of the IP chart and overall good consistency between the IP and SL charts, highlighting the potential of the IP chart for clinical and public screening applications.

One of the primary limitations of the IP chart is its lack of stability, as reflected by the broad 95% limits of agreement (−0.26 to 0.31 and −0.34 to 0.39 for right and left eye) in the consistency analysis, indicating that the IP chart misestimated visual acuity in a notable portion of participants. The discrepancy may be attributed to both technical and user-related factors. From a technical standpoint, the IP chart tended to underestimate visual acuity, particularly in patients with poorer vision, when compared to measurements obtained using the SL chart. This underestimation may be attributed to the considerable visual fatigue caused by the electronic screen of the IP chart, as reported by some participants. In terms of user factors, given that the test was fully automated and unguided, variability in results may have been greater compared to manual testing with the more familiar SL chart, where guidance from a trained examiner is typically provided. In addition, in the absence of supervision, participants may have been less motivated to perform optimally, potentially abandoning the task prematurely.

Thirunavukarasu et al. (14) investigated the accuracy, reliability, usability, and acceptability of DigiVis, a web-based application for self-testing distance visual acuity. Their study found that the bias between DigiVis measurements and standard clinical tests was −0.001 logMAR (95% CI −0.017 to 0.015), with limits of agreement (LOA) ranging from −0.175 to 0.173, and a test-retest intraclass correlation coefficient (ICC) of 0.922 (95% CI 0.887–0.946). However, the study excluded patients with visual acuity worse than +0.8 logMAR, likely due to the challenges these individuals might face in accessing the test. This exclusion may have contributed to better consistency and repeatability in the results. Zhao et al. (15) investigated the ability of the Peek Acuity smartphone application to assess visual acuity and screen for ocular conditions in children. Their study found a strong correlation between Peek Acuity and standard clinical methods, with ICCs of 0.88 (95% confidence interval 0.83–0.92) for the first eye and 0.85 (95% confidence interval 0.78–0.89) for the second eye. In contrast, our study achieved higher ICC values, indicating superior consistency and reliability in visual acuity assessments. However, this study did not report Bland–Altman statistical analyses. Bastawrous et al. (16) investigated the development and validation of the Peek Acuity smartphone-based visual acuity test for clinical practice and community-based fieldwork. Their study compared the smartphone-based Peek Acuity with Snellen acuity and the ETDRS logMAR chart in 300 adults aged 55 years and older in rural Kenya. The results indicated good agreement between Peek Acuity and the reference standards, with mean differences of 0.07 (95% CI, 0.05–0.09) logMAR for the ETDRS chart and 0.08 (95% CI, 0.06–0.10) logMAR for Snellen acuity. The study also demonstrated that Peek Acuity had a test-retest variability of ±0.029 logMAR. The visual acuity measurements using the IP chart in our study show similar repeatability, with enhanced consistency.

Subgroup analysis by age showed that good consistency still maintained in most subgroups, except for the left eyes of the 51–70 years old subgroup. We thought several factors may contribute to the poor consistency. Firstly, it is important to note that the findings in the 51–70 age group should be interpreted with caution due to the limited sample size of only 12 participants. Second, different from the IP chart which conducted VA measurements with both eyes open, left eyes were covered by an adhesive patch for a long spell before the test in the SL chart measurements. After covering, the left eyes have to make an effort to see clearly again through near-to-far accommodation, an ability that differs among people and degenerates with age (17). In addition, the crowding phenomenon was also a factor that exists in the measurements of the SL chart, but not the IP chart, as only a single optotype was displayed at one time. The crowding phenomenon increased the difficulty of identifying optotypes in the SL chart measurements, especially in the older ones (18), which can be complicated in the older age group by conditions, such as refractive errors, cataracts, fundus diseases, etc. The near-to-far accommodation and the crowding phenomenon, both of which only existed in the SL chart measurements, together may lead to the relatively low consistency of the left eyes of the 51–70 years old subgroup.

The analysis of time consumption in the consistency test suggested a learning effect of the IP chart. The second and third time of the VA test with IP chart were significantly quicker than the first time, while there was no difference between the second and the third one, indicating one single operation is enough for the examinees to be familiar with the test. Before using the IP chart, it is necessary to provide instruction and guidance to the examinee, which may increase the examination time for first-time users. No significant difference in examination time was revealed between with IP chart and the SL chart, indicating VA can be acquired efficiently with the IP chart with enough guidance.

The IP chart eliminates the learning and memory effect caused by fixed optotypes in SL charts. Setting uniform measurement rules and scoring standards programmatically reduces or eliminates interference caused by the personal habits of different examiners.

The IP chart also enables examinees to complete the test independently, thereby reducing the need for manpower. The device uses a virtual distance estimate for vision testing instead of relying on actual physical distance. This offers a more flexible, portable, and space-saving solution for vision assessment, enhancing adaptability in various clinical settings. In addition, the test is completed with both eyes open, which made it more comfortable for the examinees and more in line with real situations, as pupil size is smaller under binocular conditions compared to monocular conditions, which have an impact on VA measurements (19). The one optotype displayed one time pattern eliminates the interference of the crowding phenomenon which is a prevalent defect of the existing eye chart (20). These advantages over SL charts with traditional light boxes render promising prospects for IP charts to be used in clinical settings and public health screening. Notably, there is a height difference between the machine plane and the ground, requiring the examinee to step up onto a platform for vision testing, posing positional limitations and a risk of falling. If widespread use is anticipated, special consideration should be given to the safety of the subjects to prevent falls.

It is incumbent upon us to acknowledge the limitations inherent in this study. First, the subjects were recruited from routine ophthalmic outpatient clinics and nearby communities, and the right eyes were tested first with SL chart as a working habit, which may introduce bias. Second, the detailed records of the participants’ ocular conditions were not documented, and consequently, it is not possible to analyze the effects of certain ophthalmic diseases on VA tested with the IP chart. In addition, the cognitive state of the examinees can affect the use of the IP chart. Another limitation of this study is the small sample size within the 51–70 age group, which included only 12 participants. This limited sample may compromise the reliability of the findings for this demographic group. To address this limitation, future research should aim to increase the sample size to enable a more comprehensive evaluation of consistency within the 51–70 age group.

## Conclusion

The IP chart demonstrated good repeatability and overall consistency with the SL chart, highlighting its potential as a reliable tool for visual acuity assessment. However, some instability was observed in the measurements obtained with the new chart.

## Data Availability

The raw data supporting the conclusions of this article will be made available by the authors, without undue reservation.

## References

[1] Baker CW Josic K Maguire MG Jampol LM Martin DF Rofagha S. Comparison of Snellen visual acuity measurements in retinal clinical practice to electronic ETDRS protocol visual acuity assessment. Ophthalmology. (2023) 130:533–41. doi: 10.1016/j.ophtha.2022.12.008, PMID: 36521571 PMC10291514

[2] Wang T Huang PJ Chen C Liu DW Yi JL. A comparison of visual acuity measured by ETDRS chart and standard logarithmic visual acuity chart among outpatients. Int J Ophthalmol. (2021) 14:536–40. doi: 10.18240/ijo.2021.04.09, PMID: 33875944 PMC8025170

[3] Zhang X Zhou Y Wang Y Du W Yang J. Trend of myopia through different interventions from 2010 to 2050: findings from eastern Chinese student surveillance study. Front Med. (2022) 9:1069649. doi: 10.3389/fmed.2022.1069649, PMID: 36743682 PMC9889364

[4] Wang Q Wang C Ye T. Standard for logarithmic visual acuity charts. Beijing: Standards Press of China (2012).

[5] Wang S Hao X Ma X Yu Y Wu L Wang Y. Associations between poor vision, vision-related behaviors and mathematics achievement in Chinese students from the CNAEQ-PEH 2015. Int J Environ Res Public Health. (2020) 17:8561. doi: 10.3390/ijerph17228561, PMID: 33218140 PMC7698834

[6] Hoffer KJ Savini G. Update on intraocular lens power calculation study protocols: the better way to design and report clinical trials. Ophthalmology. (2021) 128:e115–20. doi: 10.1016/j.ophtha.2020.07.005, PMID: 32653457

[7] Cui JJ Zhang MM Luo MY Gan LY Yang JY Xie HY. Veracity of using a visual chart with a testing distance of 2.5 meters for measurement of distance visual acuity in teenagers. Zhonghua Yan Ke Za Zhi. (2021) 57:122–5. doi: 10.3760/cma.j.cn112142-20200429-00296, PMID: 33541053

[8] Thirunavukarasu AJ Hassan R Limonard A Savant SV. Accuracy and reliability of self-administered visual acuity tests: systematic review of pragmatic trials. PLoS One. (2023) 18:e0281847. doi: 10.1371/journal.pone.0281847, PMID: 37347757 PMC10286971

[9] Kuo HK Kuo MT Tiong IS Wu PC Chen YJ Chen CH. Visual acuity as measured with Landolt C chart and early treatment of diabetic retinopathy study (ETDRS) chart. Graefes Arch Clin Exp Ophthalmol. (2011) 249:601–5. doi: 10.1007/s00417-010-1461-3, PMID: 20658145

[10] Kiser AK Mladenovich D Eshraghi F Bourdeau D Dagnelie G. Reliability and consistency of visual acuity and contrast sensitivity measures in advanced eye disease. Optom Vis Sci. (2005) 82:946–54. doi: 10.1097/01.opx.0000187863.12609.7b, PMID: 16317369

[11] Lovie-Kitchin JE Brown B. Repeatability and intercorrelations of standard vision tests as a function of age. Optom Vis Sci. (2000) 77:412–20. doi: 10.1097/00006324-200008000-00008, PMID: 10966067

[12] Bhambra N Dhillon J Rahman S El-Hadad C. Development and validation of the first Canadian aboriginal syllabics visual acuity chart. Can J Ophthalmol. (2024) 59:e117–23. doi: 10.1016/j.jcjo.2023.01.009, PMID: 36796441

[13] Sailoganathan A Osuobeni EP Siderov J. A standardized logarithm of the minimum angle of resolution visual acuity chart in Hindi. Indian J Ophthalmol. (2018) 66:634–40. doi: 10.4103/ijo.IJO_1074_17, PMID: 29676304 PMC5939152

[14] Thirunavukarasu AJ Mullinger D Rufus-Toye RM Farrell S Allen LE. Clinical validation of a novel web-application for remote assessment of distance visual acuity. Eye. (2022) 36:2057–61. doi: 10.1038/s41433-021-01760-2, PMID: 34462579 PMC8403827

[15] Zhao L Stinnett SS Prakalapakorn SG. Visual acuity assessment and vision screening using a novel smartphone application. J Pediatr. (2019) 213:203–210.e1. doi: 10.1016/j.jpeds.2019.06.021, PMID: 31326117

[16] Bastawrous A Rono HK Livingstone IAT Weiss HA Jordan S Kuper H. Development and validation of a smartphone-based visual acuity test (peek acuity) for clinical practice and community-based fieldwork. JAMA Ophthalmol. (2015) 133:930–7. doi: 10.1001/jamaophthalmol.2015.1468, PMID: 26022921 PMC5321502

[17] Schaeffel F Wilhelm H Zrenner E. Inter-individual variability in the dynamics of natural accommodation in humans: relation to age and refractive errors. J Physiol. (1993) 461:301–20. doi: 10.1113/jphysiol.1993.sp019515, PMID: 8350267 PMC1175259

[18] Liu R Patel BN Kwon M. Age-related changes in crowding and reading speed. Sci Rep. (2017) 7:8271. doi: 10.1038/s41598-017-08652-0, PMID: 28811585 PMC5557829

[19] Martino F Pereira-da-Mota AF Amorim-de-Sousa A Castro-Torres JJ González-Méijome JM. Pupil size effect on binocular summation for visual acuity and light disturbance. Int Ophthalmol. (2023) 43:2183–95. doi: 10.1007/s10792-022-02614-w, PMID: 36512297

[20] Facchin A Maffioletti S Martelli M Daini R. Different trajectories in the development of visual acuity with different levels of crowding: the Milan Eye Chart (MEC). Vis Res. (2019) 156:10–6. doi: 10.1016/j.visres.2019.01.003, PMID: 30639454

